# Supplementary material for: Laser Micropatterning Promotes Rete Ridge Formation and Enhanced Engineered Skin Strength without Increased Inflammation
Source: Bioengineering (Basel). 2023 Jul 20;10(7):861. doi: 10.3390/bioengineering10070861 (PMC10376754; doi:10.3390/bioengineering10070861)
Supplement: Supplementary file 1 [file bioengineering-10-00861-s001.zip › bioengineering-2470780-supplementary.pdf]

| **Table S1.** Product numbers for $RT^2$ qPCR Primer Assays (purchased from Qiagen, Inc.). | | | |
|---|---|---|---|
| **Gene Name** | **Gene Symbol** | **National Center For Biotechnology Information (NCBI) Reference Sequence #** | **Qiagen Product Number (GeneGlobe ID #)** |
| Glyceraldehyde 3-phosphate dehydrogenase | GAPDH | NM_002046 | PPH00150F-200 |
| Interleukin 6 | IL6 | NM_000600 | PPH00560C-200 |
| Interleukin 8 (C-X-C motif chemokine ligand 8) | IL8 (CXCL8) | NM_000584 | PPH00568A-200 |
| Monocyte Chemoattractant Protein-1 (C-C motif chemokine ligand 2) | MCP-1 (CCL2) | NM_002982 | PPH00192F-200 |
| Heat Shock Protein 47 (serpin family H member 1) | HSP47 (SERPINH1) | NM_001235 | PPH01194C-200 |
| Heat shock protein family A (Hsp70) member 1A | HSP70 (HSPA1A) | NM_005345 | PPH01193B-200 |
| Heat shock protein family A (Hsp70) member 1B | HSP72 (HSPA1B) | NM_005346 | PPH01216B-200 |
| Podoplanin | PDPN | NM_006474 | PPH22397A-200 |
| Decorin | DCN | NM_001920 | PPH01900A-200 |
| Transglutaminase 2 | TGM2 | NM_004613 | PPH23362A-200 |
| Versican | VCAN | NM_004385 | PPH06098D-200 |

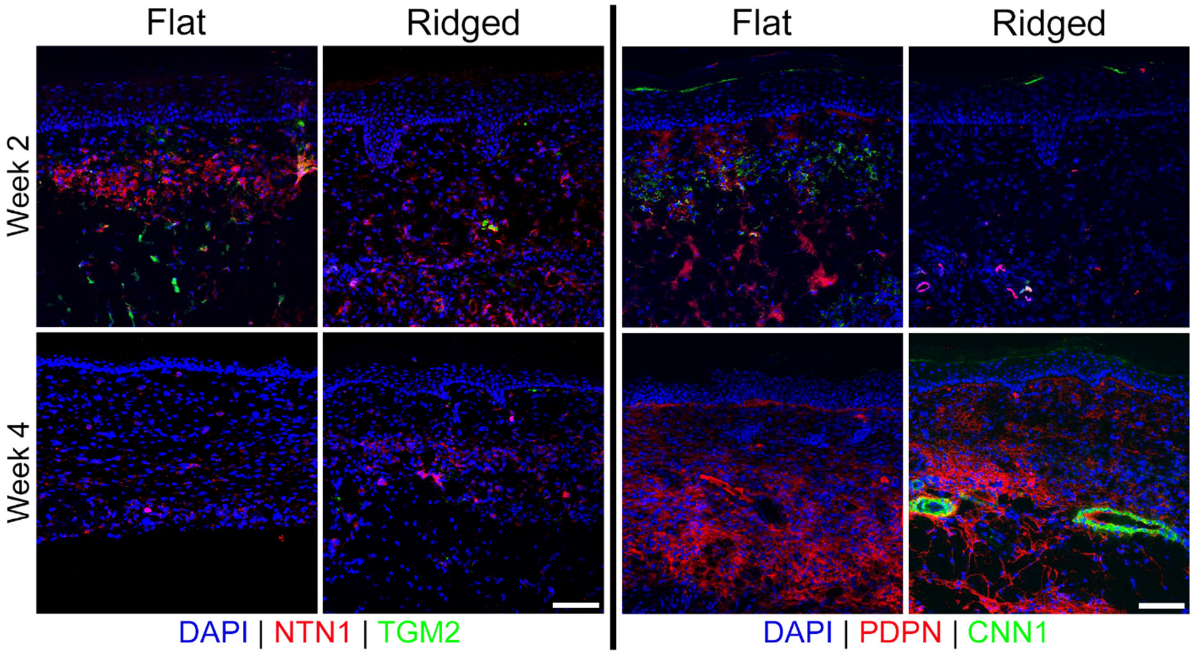


Figure S1: Immunohistochemical localization of papillary (NTN1 and PDPN; red) and reticular (TGM2 and CNN1; green) fibroblast-associated markers in Flat and Ridged grafts at 2 and 4 weeks post-grafting. Nuclei were counterstained using DAPI (blue). Both sets of markers were observed in Flat grafts at week 2, while mainly only papillary-associated markers were found in Ridged grafts at week 2 and both groups at week 4. CNN1 was found in Ridged grafts at week 4, though it was limited to blood vessels. Scale bar = 100 µm.

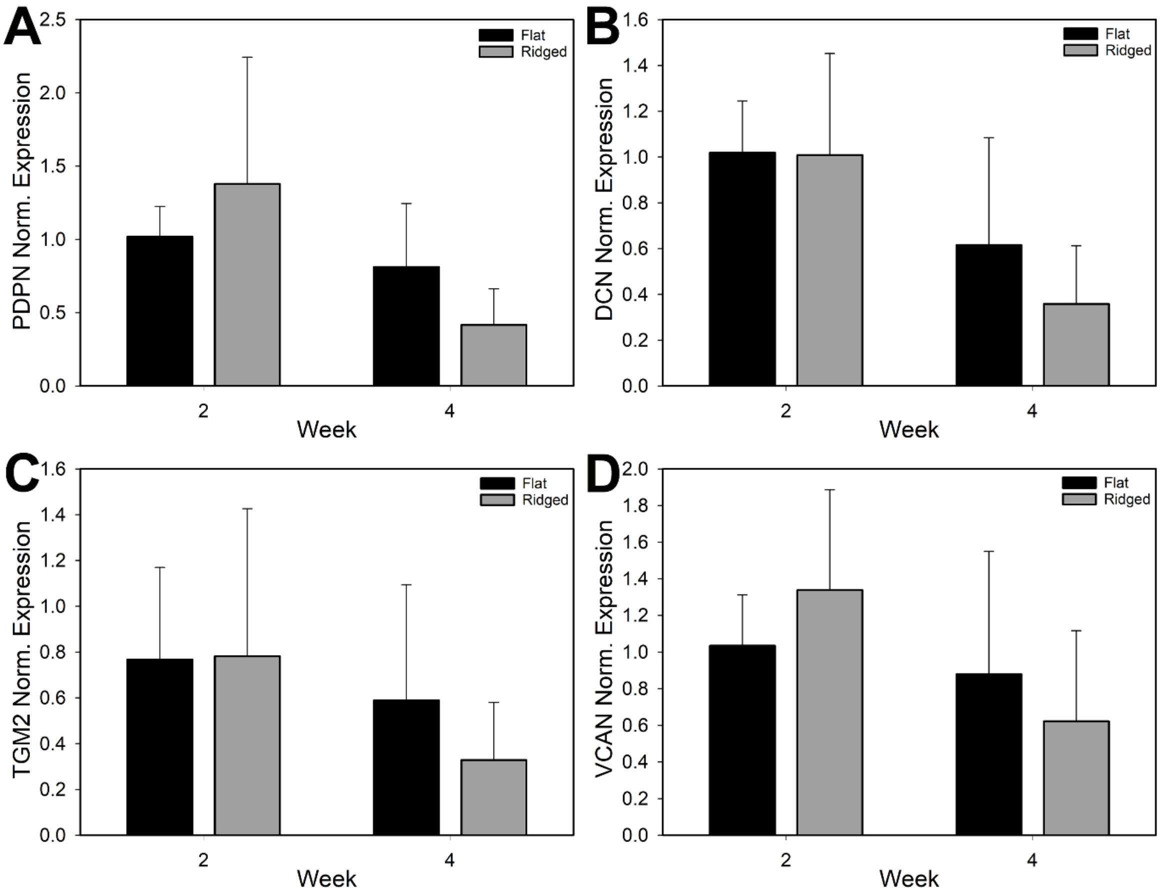


Figure S2: Gene expression analysis for genes associated with papillary dermal fibroblasts (PDPN, A) and ECM (DCN, B) and genes associated with reticular dermal fibroblasts (TGM2, C) and ECM (VCAN, D) in Flat and Ridged grafts at 2 and 4 weeks post-grafting. Expression was referenced to GAPDH presence and normalized to mean expression in Flat grafts at week 2. No statistical differences were determined between the groups at either time point ($p>0.05$).
